# *In Vivo* Disruption of TGF-β Signaling by Smad7 in Airway Epithelium Alleviates Allergic Asthma but Aggravates Lung Carcinogenesis in Mouse

**DOI:** 10.1371/journal.pone.0010149

**Published:** 2010-04-13

**Authors:** Xiaolin Luo, Qiurong Ding, Min Wang, Zhigang Li, Kairui Mao, Bing Sun, Yi Pan, Zhenzhen Wang, Ying Qin Zang, Yan Chen

**Affiliations:** 1 Key Laboratory of Nutrition and Metabolism, Institute for Nutritional Sciences, Chinese Academy of Sciences, Shanghai, China; 2 Laboratory of Molecular Cell Biology, Institute of Biochemistry and Cell Biology, Shanghai Institutes for Biological Sciences, Chinese Academy of Sciences, Shanghai, China; Sun Yat-Sen University, China

## Abstract

**Background:**

TGF-β has been postulated to play an important role in the maintenance of epithelial homeostasis and the development of epithelium-derived cancers. However, most of previous studies are mainly focused on the function of TGF-β in immune cells to the development of allergic asthma and how TGF-β signaling in airway epithelium itself in allergic inflammation is largely unknown. Furthermore, the *in vivo* TGF-β function specifically in the airway epithelium during lung cancer development has been largely elusive.

**Methodology/Principal Findings:**

To evaluate the *in vivo* contribution of TGF-β signaling in lung epithelium to the development of allergic disease and lung cancer, we generated a transgenic mouse model with Smad7, an intracellular inhibitor of TGF-β signaling, constitutively expressed in mouse airway Clara cells using a mouse CC10 promoter. The mice were subjected to the development of OVA-induced allergic asthma and urethane-induced lung cancer. The Smad7 transgenic animals significantly protected from OVA-induced asthma, with reduced airway inflammation, airway mucus production, extracellular matrix deposition, and production of OVA-specific IgE. Further analysis of cytokine profiles in lung homogenates revealed that the Th2 cytokines including IL-4, IL-5 and IL-13, as well as other cytokines including IL-17, IL-1, IL-6, IP10, G-CSF, and GM-CSF were significantly reduced in the transgenic mice upon OVA induction. In contrast, the Smad7 transgenic animals had an increased incidence of lung carcinogenesis when subjected to urethane treatment.

**Conclusion/Significance:**

These studies, therefore, demonstrate for the first time the *in vivo* function of TGF-β signaling specifically in airway epithelium during the development of allergic asthma and lung cancer.

## Introduction

Airway epithelium functions as a complex physical barrier that defends against exposure to potentially harmful inhaled substances and microbial pathogens. It is now believed that airway epithelial cells also play a central role in innate and adaptive immune response as well as mucosal inflammation that are closely integrated into the development of allergic airway diseases such as asthma [1]. Airway epithelial cells produces host defense molecules, cytokines and chemokines upon activation of pathogen recognition receptors such as toll-like receptors (TLRs). Epithelium-derived cytokines recruits dendritic cells, T cells and B cells into close proximity of epithelium to mediate adaptive immune response through interaction with epithelial cells. On the other hand, epithelial cells can serve as a target for immune cells, implicated in the immune response and mucus production in inflammatory airway diseases. Therefore, further investigation of functions of epithelial cells in the immune and inflammatory responses can not only aid in understanding the pathophysiological basis of airway diseases, but also help for the future therapeutical invention of these diseases.

TGF-βs belong to a widely expressed family of cytokines with pleiotropic effects on a variety of cellular functions such as cell growth, proliferation, differentiation, and apoptosis. TGF-β signaling starts by binding of ligand to the cognate transmembrane receptor kinase, followed by activation of Smad proteins that transduce the signal from the plasma membrane into the nucleus where Smad and its transcriptional partners directly regulate gene expression [2], [3]. According to their functional and structural features, Smads are classified into receptor-specific Smads (R-Smads), a common-Smad (Co-Smad or Smad4), and inhibitory Smads (I-Smads) [3], [4].Smad7 is a member of the I-Smad subfamily that is able to antagonize TGF-β signaling by direct interaction with the type I receptor [5]. Previous studies have indicated that TGF-β is implicated in the regulation of airway inflammation and hyperresponsiveness in asthma development. Measurement of immunoreactive TGF-β1 in bronchoalveolar lavage fluid revealed that the basal TGF-β1 level is significantly elevated in atopic asthma patients and it increases further in response to allergen exposure.[6] Th2-dominated inflammation is well illustrated to be the cornerstone of the disorder [7], with airway remodeling believed to be an adverse consequence of the inflammatory response [8]. TGF-β secreted by T cells was found to play an important role in the down-modulation of the immune response to high doses of antigen [9]. Th cells engineered to express latent TGF-β abolished airway hyperreacivity and airway inflammation induced by OVA-specific Th2 effector cells in SCID and BALB/c mice [10]. And transgenic mice with selective expression Smad7 in mature T cells to block TGF-β signaling in T cells enhanced airway inflammation and airway reactivity [11] However, most of these studies are mainly focused on the function of TGF-β in immune cells to the development of asthma. How TGF-β signaling in airway epithelium itself is implicated in allergic inflammation is largely unknown.

In addition to immune cells, marked activation of epithelial cells was another important characteristics of allergic diseases and substantial evidence has demonstrated that epithelial cells are able to mediate and regulate both innate and adaptive immune responses [12], indicating that malfunction of epithelia cells is involved in the initial cause of allergic diseases. The widespread expression pattern of TGF-βs in epithelial cells suggests that this family of peptides may play important roles in the maintenance of epithelial homeostasis. Previous study revealed that expression of Smad7 in bronchial epithelial cells is inversely correlated to basement membrane thickness and airway hyperresponsiveness in patients with asthma [13]. In the asthmatic airway, a positive correlation between epithelial damage and airway hyperresponsiveness has been observed [14], while imbalance between proliferation and apoptosis of epithelial cells was suggested to cause an increased loss of parts of the epithelial layer [15]. TGF-β signaling has previously been shown to induce apoptosis in airway epithelial cells that indicate TGF-β contributes to the airway epithelial layer damage in the asthmatic airway [16] However, the exact function of TGF-β in airway epithelium other than induction of apoptosis during the development of allergic asthma remains unclear. To better understand whether or not TGF-β signaling in epithelial cells contributes to asthma development, we expressed Smad7 in mouse airway progenitor cells using a mouse Clara cell specific 10 kDa protein (CC10) promoter to specifically block TGF-β signaling pathway in airway epithelial cells. Using the OVA-induced asthma model, we analyzed the role of Smad7 in the development of allergic asthma.

Besides allergic diseases, the other type of severe lung disease is cancer, which is thought to be a direct consequence of epithelium malfunction. Alterations in TGF-β signaling have significant effects on tumor initiation and progression. The function of TGF-β as a tumor suppressor or tumor promoter dependent on the context and stage of tumor progression have been carefully characterized [17]. However, the specific role of TGF-β signaling in airway epithelial cells in lung cancer progression has been largely elusive. To better characterize the biological functions of TGF-β signaling in Clara cells in the development of lung cancer, we subjected the CC10-Smad7 transgenic mouse with urethane treatment to induce lung cancer and investigated how TGF-β signaling in Clara cells might contribute to the development of lung cancer, in addition to its effect in the development of allergic asthma.

## Results

### Generation and characterization of CC10-Smad7 transgenic mice

**Figure 1:**
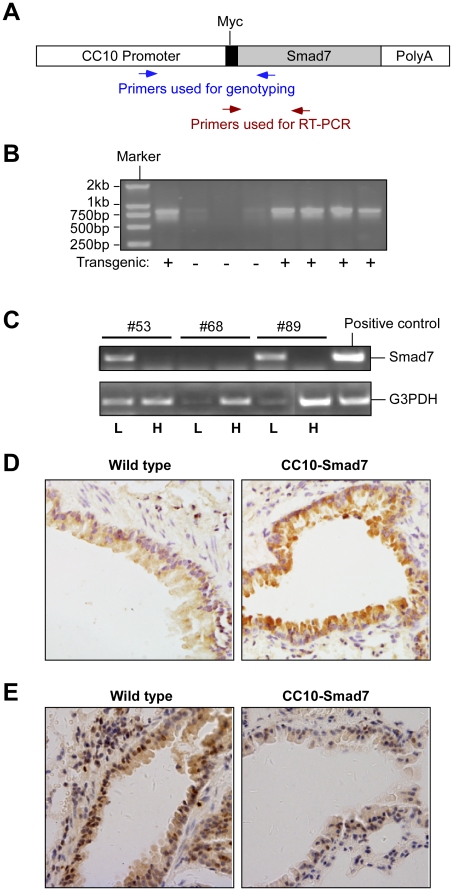
Generation of transgenic mouse. (A) A diagram depicting the transgenic construct. A mouse CC10 promoter was placed upstream of a rat Smad7 cDNA gene. A Myc tag was placed at the N-terminal end of the Smad7 transcript. The relative positions of PCR primers are indicated. (B) Genotyping of transgenic mice by PCR. Genomic DNA isolated from mouse tails was used in genomic PCR with a pair of primers specific for the Smad7 transgene, giving rise to a product of 843 bp. A representative result is shown here with five positive samples marked with “+”. (C) Expression of Smad7 transgene mRNA in the mouse lung. Total RNA was isolated from mouse tissues, including lung (L) and heart (H). RT-PCR was performed to detect the expression of Myc tagged-Smad7 with a pair of primers specific for transgene. Expression of G3PDH (glyceraldehyde-3-phosphate dehydrogenase) was used as quantity control of cDNA template. A representative result is shown here with two positive samples (#53 and #89) and a negative sample (#68). The Myc-tagged Smad7 plasmid was used as template for a positive control. (D) Analysis of the Smad7 by immunohistochemistry staining. Representative lung sections (400×) from wild type and the CC10-Smad7 transgenic mice were used in immunohistochemistry staining with an anti-Smad7 antibody. The nuclei were stained with haematoxylin. Note the increased expression of Smad7 in bronchiolar epithelium but not in other cells. (E) Disruption of TGF-β signaling by Smad7 in the transgenic mouse. Representative lung sections (400×) were used in immunohistochemistry staining with an antibody for phosphorylated Smad2 antibody. The nuclei were stained with haematoxylin. Note the decreased phosphorylation level of Smad2 in bronchiolar epithelium in the transgenic mouse.

Smad7 is an inhibitory Smad protein that is able to bind TGF-β type I receptor and block TGF-β signaling in the cells [5]. To address the functional significance of TGF-β signaling in airway epithelium in allergic airways disease and lung carcinogenesis, we disrupted the *in vivo* TGF-β signaling by exogenous expression of Smad7 specifically in the airway epithelium. We generated a transgenic construct by placing Smad7 under the transcriptional control of CC10 promoter (CC10-Smad7) to express Smad7 specifically in Clara cells, which are postulated to be pivotal in forming the cell lineage of the bronchiolar epithelium [18]. A Myc-tag was constructed at the N-terminus of Smad7 transcript to facilitate detection of the transgene expression in the animals (Figure 1A). Previous study showed that the inhibitory activity of Smad7 was not affected by this Myc-tag. [19]. The plasmid construct was linearized and used in microinjection to generate the transgenic mice. The mouse founders were identified by genotyping using genomic DNA isolated from the mouse tails (Figure 1B). From a total of 83 offspring in the B6CBF1 background given by the pseudopregnant foster mothers, 8 founders were identified to carry the transgene. All mice positive for the transgene showed no signs of health problems up to 1 year of age as compared with the wild type littermates.

To verifying Smad7 transgene expression, RT-PCR was first performed to detect the expression of exogenous Smad7 with a pair of primers specific for the transgene as shown in Figure 1A. The mRNA of the Myc-tagged Smad7 was only detected in the lung of the transgenic mice, but neither in the lung of the wide type mice nor in the heart of the transgenic mice (Figure 1C). The expression of Smad7 protein was later examined by immunohistochemistry staining in the lung sections. As a result, we found that the protein level of Smad7 was markedly increased in airway bronchiolar epithelium in transgenic mice in comparison with the wide type animals (Figure 1D). The mice positive for the Smad7 transgene were crossed with C57BL/6J strain to generate transgenic mice used in this study. Therefore, all the mice used in the analyses were 50% B6CBF1 and 50% C57BL/6J in their genetic background. To confirm that expression of the exogenous Smad7 is able to block TGF-β signaling, we performed immunohistochemistry staining to detect the phosphorylation of Smad2, which is one of the major signaling events after TGF-β receptor activation[3], [4]. As expected, phosphorylation of Smad2 was largely decreased in the Smad7 transgenic animals specifically in epithelium (Figure 1E).

### Overexpression of Smad7 in airway epithelium alleviates allergic inflammation and airway remodeling

**Figure 2:**
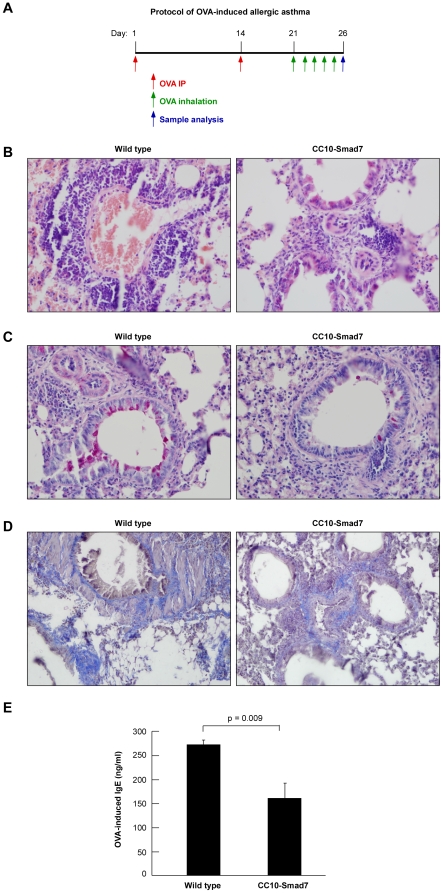
Overexpression of Smad7 in airway epithelium alleviates allergic inflammation and airway remodeling. (A) Schematic presentation of the study protocol for OVA-induced allergic asthma. Airway inflammation and remodeling was induced in female C57BL/6J mice sensitized with OVA in alum, and subjected to serial aerosolized OVA. (B) Reduced airway inflammation in OVA-sensitized and challenged CC10-Smad7 mice. Representative sections from paraffin-embedded lung were stained using H&E. Note that the inflammatory cells recruitment was largely reduced in CC10-Smad7 mice. The magnification of the image is 400×. (C) Reduced airway mucus production in OVA-induced asthma in CC10-Smad7 mice. Representative sections (400×) from paraffin-embedded lung were stained using PAS. Note that that the mucus stained by PSA (reddish-purple) produced by airway cells was reduced in CC10-Smad7 mice. (D) Decreased airway extracellular matrix deposition in OVA-induced asthma in CC10-Smad7. Representative sections (400×) from paraffin-embedded lung were stained using MSB. Note the peribronchial fibrosis was decreased in CC10-Smad7 mice. (E) Reduced OVA-specific IgE concentration in CC10-Smad7 mice. The mouse serum was analyzed for OVA-specific IgE by ELISA. Values are mean ± SD from five mice per group.

A majority of previous studies have demonstrated that TGF-β serves as a negative regulator of airway inflammation and hyperresponsiveness through its regulation on immune cells [9], [10], [11], [13], [20], [21]. However, the specific role of TGF-β signaling in airway epithelium in the development of asthma remains unknown. To address the issue, both the CC10-Smad7 transgenic mice and the wild type littermates were induced to develop allergic asthma by OVA sensitization and challenge (Figure 2A).

One of the major factors in the development of allergen-induced lung pathophysiology is inflammatory cell infiltration to the airways. We determined the extent of leukocyte infiltration to the lung tissue by examination of H&E-stained histological sections. In wild type mice, OVA induction led to widespread peribronchiolar inflammation (Figure 2B, left panel). However, the inflammatory cell recruitment in CC10-Smad7 transgenic mice was dramatically decreased (Figure 2B, right panel). Prolonged allergen challenge of sensitized mice results in increased extracellular matrix deposition in bronchiolar subepithelial regions [22]. We next analyzed the function of Smad7 overexpression on extracellular matrix deposition in lung sections by MSB staining. It appeared that matrix deposition in the Smad7 transgenic mice was markedly decreased in comparison with the wide type mice (Figure 2D). Consistently, PAS staining also revealed that the mucus secretion was significantly reduced in the Smad7 transgenic mice (Figure 2C). The production of OVA specific IgE was also significantly decreased in the Smad7 transgenic mice in comparison with the wild type control (Figure 2E). Collectively, these data indicate that Smad7 expression in airway epithelial cells was able to alleviate local inflammation, matrix deposition and mucus secretion in OVA-induced allergic asthma.

### Overexpression of Smad7 in airway epithelium modulates cytokine profile upon OVA induction

**Figure 3:**
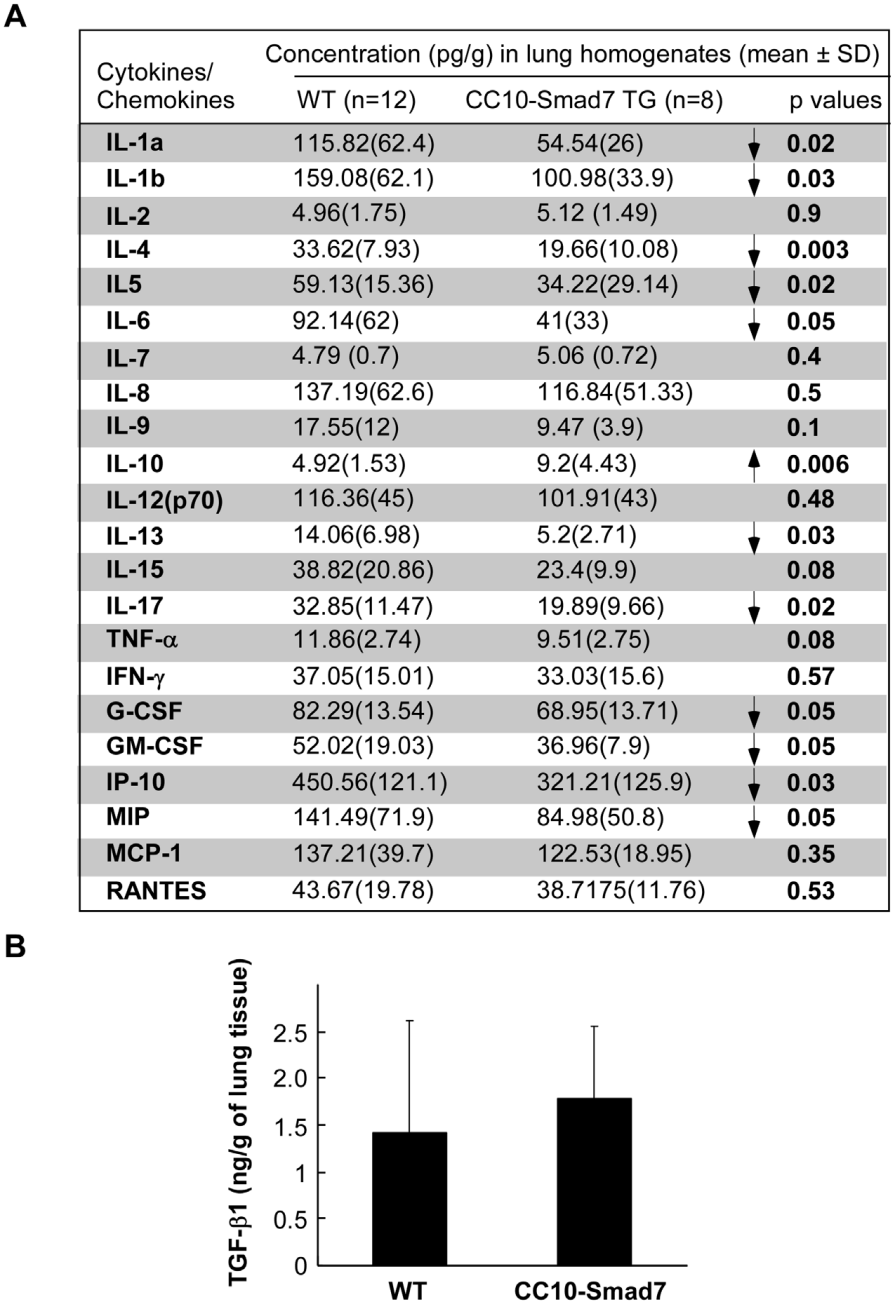
Alteration of cytokines in lung homogenate. (A) Changes of cytokines in lung homogenate. Lung homogenate was prepared from the wild type and CC10-Smad7 mice after OVA-induced asthma formation. Values are shown as means ± SD. The p-values are also shown. (B) TGF-β level in lung homogenate. Lung homogenate was prepared from the wild type and CC10-Smad7 mice after OVA-induced asthma formation. Values are shown as means ± SD.

Cytokines were found to play a critical role in orchestrating, perpetuating and amplifying the inflammatory response in asthma. The increased and abnormal expression of cytokines in airway cells is one of the major targets of corticosteroid therapy that is by far the most effective treatment for asthma currently available. To further investigate the inflammatory response in OVA-induced asthma, we analyzed the cytokine profile of the lung homogenates by ELISA method (Figure 3A). It has been shown that IL-4, IL-5 and IL-13, the well-defined Th2 cytokines, play an important role in the pathophysiology of allergic diseases including asthma [23], [24] These Th2 cytokines were significantly decreased in CC10-Smad7 mice compared to the wild type animals. Meanwhile, the Th1 cytokines such as IL-2 and IFN-γ had little change between the two groups. Interestingly, some other cytokines such as IL-1 and IL-6 that were found to play important roles in many inflammatory diseases such chronic obstructive pulmonary disease, inflammatory bowel disease and rheumatoid arthritis, were also decreased in CC10-Smad7 mice. In contrast, IL-10, a pleiotropic cytokine that has the potential to downregulate both Th1- and Th2-driven inflammatory processes [25], and has been shown to possess a beneficial effect on airway remodeling [26], [27], was significantly increased in CC10-Smad7 mice. MIP-1α, one of the downstream target of NFκB signaling [28], was decreased in the Smad7 transgenic mice, indicating a possible downregulation of NFκB signaling in the transgenic animals. GM-CSF, a key cytokine for eosinophil survival and being produced by both Th2 and Th1 cells as well as epithelial cells [29], [30], was also decreased in the Smad7 transgenic mice, consistent with the previous finding that neutralization of GM-CSF with antibodies could attenuate airway hyper-responsiveness in a murine asthma model [31]. Similar to GM-CSF, another neutrophil factor, G-CSF, was significantly decreased in CC10-Smad7 transgenic mice. IP-10, which was found to be involved in human allergic pulmonary reaction and increased in asthmatic children[32], [33], was also decreased in the transgenic mice, indicating that the cytokine secretion was extensively changed toward a favorite direction in the transgenic animals. Another cytokine, IL-17, which was found to be correlated with the degree of severity of airway hypersensitivity in asthmatic patients [34], was also significantly reduced in CC10-Smad7 mice (Figure 3A). Further analysis revealed that TGF-β1 concentration was not significantly changed in CC10-Smad7 mice (Figure 3B). Collectively, these data not only confirm that disruption of TGF-β signaling in the lung epithelium is able to alleviate OVA-induced allergic response in the lung, but may also indicate that alteration of TGF-β signaling in the epithelium itself is able to modulate cytokine secretion by either the epithelial cells or the immune cells recruited to the epithelium.

### Overexpression of Smad7 in airway epithelium increased the incidence of lung carcinogenesis following exposure to urethane

**Figure 4:**
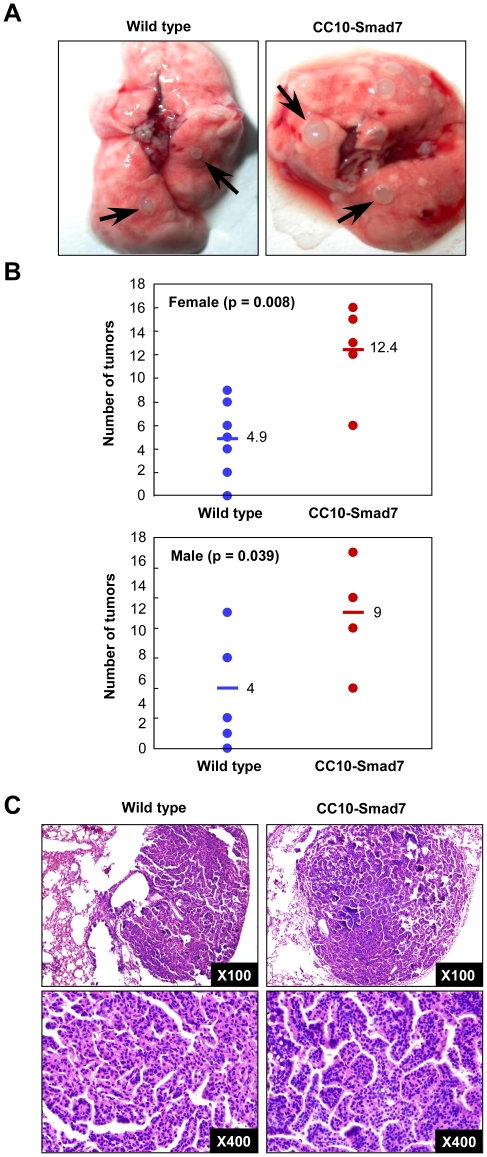
Overexpression of Smad7 in airways increased the lung carcinogenesis following exposure to Urethane. (A) Adenomas (shown by arrows) are visible on the surface of the lungs of wild type and CC10-Smad7 mice following exposure to urethane. Note that the number of adenoma in the Smad7 transgenic mice is substantially increased. (B) Average number of tumors per mouse was largely increased in both male and female CC10-Smad7 transgenic mice. The bars shown are average numbers in each group. (C) Gross histology of the lung tumors found in wild type and CC10-Smad7 mice following urethane exposure.

Alterations in TGF-β signaling are linked to a variety of human diseases, including cancers[17]. The disruption of TGF-β signaling occurs in several human cancers and it has been proposed that TGF-β possesses a tumor suppressor function in the initial phase of cancer development[17]. Smad6 and Smad7 are two inhibitory Smad proteins, while Smad7 is relatively specific for blocking TGF-β signaling and Smad6 is specific for blocking BMP signals. Smad6 was recently found to contribute to patient survival in non-small cell lung cancer [35]. However, the contribution of Smad7 specifically in lung epithelium to lung cancer has not been characterized. In this study, we analyzed whether disruption of TGF-β signaling specifically in the lung epithelium is implicated in the development of lung cancer. To address this issue, we subjected both the wild type and CC10-Smad7 mice to urethane treatment. Five months after urethane treatment, the mice were sacrificed and the tumor formation in the lung was analyzed. In contrast to the protective role of Smad7 on asthma development, CC10-Smad7 mice showed significantly increased incidence of lung tumors in comparison with the wild type controls (Figure 4A). Statistic analysis revealed that the tumor number in CC10-Smad7 mice was about twice of those in the wild type mice in both male and female animals (Figure 4B). Except for the changes of tumor incidence, there appeared no discernable difference in the structural property of the tumor as histological analysis revealed the same type of adenocarcinoma in both the wide type and Smad7 transgenic mice (Figure 4C).

## Discussion

In this study, we specifically blocked TGF-β signaling in airway epithelium by overexpression Smad7 in Clara cells. The contribution of TGF-β signaling specifically in Clara cells in allergic asthma and lung cancer was investigated. Using the transgenic mouse model, our studies uncover for the first time the important function of TGF-β signaling in airway epithelium itself in the development of allergic asthma and lung cancer. Blocking TGF-β signaling in Clara cells appears to have a protective role in OVA-induced asthma, while it can increase the incidence of urethane-induced lung cancer.

Allergic asthma is a complex disorder and the pathogenesis of this disease can be regarded as a two-step phenomenon. The first step consists of sensitization to an aeroallergen and preferential activation of antigen specific Th2 cells. The second step involves targeting the Th2-driven allergic inflammation to the lower airway. The inflammatory process is orchestrated and regulated by a complex network of mutually interacting cytokines and growth factors, secreted not only by a range of inflammatory cells but also by structural tissue components including epithelial cells, fibroblasts and smooth muscle cells, subsequently leading to remodeling of airway wall. In OVA-induced asthma model, CC10-Smad7 transgenic mice showed reduced airway inflammation characterized by decreases in immune cell infiltration, extracellular matrix deposition, mucus production, and production of OVA-specific IgE. The reduced airway mucus production and extracellular matrix deposition in the Smad7 transgenic mice revealed a slowed progression in airway remodeling. Further analysis of several cytokines in the lung homogenates demonstrated that the local immune response was significantly alleviated in the transgenic animals.

Our data uncover for the first time that specifically blocking the TGF-β signaling by overexpression Smad7 in lung epithelium, but not in immune cells, is sufficient to relieve the development of allergic asthma. Previous studies were mainly focused on the actions of TGF-β in immune cells in the pathophysiology of asthma. For example, selective expression Smad7 in mature T cells to block TGF-β signaling in T cells could enhance airway inflammation and airway reactivity [11]. However, in comparison with blocking TGF-β signaling in T cells, our results demonstrated an opposite action of TGF-β pathway in regulating airway inflammation and expression of many pro-inflammatory cytokines when TGF-β signaling is selectively blocked in Clara cells. We postulate that blocking TGF-β signaling pathway specifically in lung epithelium will affect the epithelium-mediated adaptive immune response, such as cytokine secretion to recruit immune cells into the proximity of epithelium during the progression of inflammatory response. Our data, therefore, reveal that TGF-β signaling pathway has a different function in regulating airway inflammation dependent on the context of cell type. In addition, previous studies have been targeting on the effect of TGF-β signaling on epithelium apoptosis[16]. However, our data demonstrate that there is no significant change in cell apoptosis between the wild type and CC10-Smad7 transgenic mice (Figure S1), indicating that apoptosis may not play a primary role in TGF-β-mediated regulation on airway inflammation.

Our analyses with the cytokine profile in the lung homogenates provided additional evidence that TGF-β signaling in the epithelium itself is implicated in the development of allergic response. We found that the well-defined Th2 cytokines, such as IL-4, IL-5 and IL-13, were significantly decreased in the Smad7 transgenic mouse, indicating that the Th2-driven allergic inflammation is alleviated after disruption of TGF-β signaling in airway epithelium. It can be postulated that there exists a positive feedback between the activation of epithelial cells and the Th2 cells, and TGF-β signaling in epithelial cells is implicated in such positive feedback. Airway epithelial cells can undergo remodeling changes and contribute to inflammation by producing proinflammatory molecules. Indeed, epithelial cells can secrete an array of proinflammatory molecules that modulate the recruitment and functioning of immune and inflammatory cells [1]. As cytokines play a critical role in orchestrating, perpetuating and amplifying the inflammatory response in asthma, we hypothesize that the secretion of proinflammatory cytokines, and/or the positive feedback between epithelial cells and Th2 cells, are partly controlled by TGF-β signaling in epithelial cells.

In addition to Th2 cytokines, some other cytokines such as IL-1 and IL-6 that have been found to play a role in many inflammatory diseases, were also reduced in the Smad7 transgenic mice, indicating that TGF-β signaling in the airway epithelium has a general effect to modulate local inflammation. Interestingly, we found that IL-10 is increased in the lung homogenate of CC10-Smad7 mice. Consistently, IL-10 has been shown to reduce both Th1- and Th2-driven inflammatory processes [25]. Some other cytokines that were previously found to be related to asthma development such as G-CSF, GM-CSF and IP-10, had consistent change toward a favorite direction in the Smad7 transgenic mice, indicating a reduced allergic development. To our surprise, IL-17, that was found to be correlated with the degree and severity of airway hypersensitivity in asthmatic patients [34], was markedly reduced in the CC10-Smad7 mice. IL-17 is secreted by Th17 cells that comprise a recently recognized subset of T cells mediating immunity to extracellular organisms and being implicated in several autoimmune diseases [36]. The role of IL17 in animal model of allergic airway inflammation has been recently investigated in several studies[37], [38]. A dual role has been found for IL17 and the precise role of IL17 in allergic airway inflammation is not fully clear. Our study provided a piece of clue that the secretion of IL-17 may be regulated by TGF-β signaling in epithelial cells in the development of allergic asthma. The missing link between TGF-β signaling in epithelial cells and secretion of IL-17 by Th17 cells would be an interesting and important issue to investigate in the future.

In contrast to the protective role of Smad7 overexpression in OVA-induced asthma, blocking TGF-β signaling by overexpression Smad7 specifically in Clara cells was sufficient to increase the incidence of lung carcinogenesis following exposure to urethane. This result is consistent with numerous previous reports demonstrating that TGF-β signaling serves as a tumor suppressor function especially at the initial stage of carcinogenesis. Adenocarcinoma is the most common form of human lung cancer, one of the leading causes of cancer-related deaths worldwide, especially in the United States [39]. Most adenocarcinomas arise in the alveolar and bronchiloar epithelia from type II pneumocytes and Clara cells, and it is mainly those of Clara cells origin that progress to malignancy.[40]. Whether or not Clara cells have a similar etiologic function in mouse models of adenocarcinoma is controversial [41]. Our studies for the first time has highlighted the contribution of TGF-β signaling specifically in Clara cells to the development of lung adenocarcinoma in the mouse, meanwhile providing another proof indicating the important etiologic function of Clara cells in the development of adenocarcinoma. Therefore, the CC10-Smad7 mouse may be used as a useful model to further elucidate the functional role of Clara cells, especially the TGF-β signaling in Clara cells, in the development of lung adenocarcinoma in the future.

The relationship between inflammation and cancer has been broadly investigated and the general consensus is that chronic and persistent inflammation may contribute to cancer development [42], [43]. However, this is a controversial issue, especially during the early phases of carcinogenesis when innate response is beneficial and likely involved in the activation of effective surveillance by adaptive immunity to eliminate the affected cells[44], [45]. Genetic and bioinformatic analyses revealed that oncogene-induced cellular senescence (OIS), a potent cancer-protective response to oncogenic events, is linked specifically to the activation of an inflammatory transcriptome [46], [47]. IL-6 and IL-8 have been found to be required for the execution of OIS [46], [47]. In certain human tumors, the presence of an effective immune response is associated with good prognosis [48], [49]. As our result showed that the inflammation response in OVA-induced asthma was dramatically decreased in CC10-Smad7 mice, we suggest that the increased incidence of lung carcinogenesis induced by urethane treatment in these mice may partly due to the alteration of inflammatory status. However, the underlying mechanism to comprehend the contribution of TGF-β signaling in epithelium to allergic disease and cancer development especially the interplay between allergic response and cancer development needs further investigation in the future. Nevertheless, the CC10-Smad7 mouse may serve as a useful model to elucidate these important issues.

## Materials and Methods

### Ethics Statement

All animals were maintained and used in accordance with the guidelines of the Institutional Animal Care and Use Committee of the Institute for Nutritional Sciences, Shanghai Institutes for Biological Sciences, Chinese Academy of Sciences.

### Generation of CC10-Smad7 mice

The Smad7 transgene was constructed by standard recombinant DNA techniques by fusing a mouse CC10 promoter (2.3 kb) with a Myc-tagged rat Smad7 cDNA (Figure 1A). The mouse CC10 promoter was cloned from the mouse genomic DNA using primers: 5′-TTGCAGGGAGTAAGGAGGGTCATGGGA-3′ and 5′-GGCTTTGGTGTCTGTAGATGTGGGGTG-3′. The linearized transgene was used in microinjection into fertilized mouse eggs at the Model Animal Research Center, Nanjing University, Nanjing, China. All transgenic mice were generated with B6CBF1 and maintained with the C57B6/6J strain.

### Analysis of CC10-Smad7 transgene expression

Genomic DNA was extracted from a 2-mm tail biospy with a Genomic DNA Extraction protocol as described [50]. PCR genotyping primers were 5′-TCGTTGGAGGGAGGCAATAGAAGGA-3′ and 5′-GCGAGGA GGCGAGGAGAAAAGTCGT-3′. To detect the mRNA expression of the Smad7 transgene, mice were sacrificed and the lung and other tissues were removed and washed with cold PBS, homogenized, and subjected to Trizol RNA abstraction. The RNA was reverse-transcribed as described [51], and the expression of myc-Smad7 was examined by PCR with primers 5′-GAATGAAATGGAGAGCTTGGGCGAC-3′ and 5′-GAGGATGGGGG ATGGGGATGGTGGT- 3′.

### Histological analysis

Mouse lung tissues were fixed in 4% polyformaldehyde solution at 4°C overnight, and then embedded in paraffin. Paraffin sections of 4 µm were used for hematoxylin and eosin (HE) staining and immunohistochemistry (IHC). The antibodies were diluted as follows: phosphorylated Smad2 (1∶200) (from Cell Signaling Technology, Danvers, MA, USA), and Smad7 (1∶200) (from Santa Cruz, CA, USA). TUNEL assay was carried out using ApopTag® Peroxidase In Situ Apoptosis Detection Kit (from Chemicon, Temecula, CA, USA) following the instructions.

### PAS and MSB staining

PAS and MSB staining was performed as previously described [52]. For PAS staining, the mouse lung specimens were fixed in Bouin's solution overnight at room temperature and embedded in paraffin using standard techniques. The paraffin-embedded sections (4 µm) were oxidized in 0.5% periodic acid for 15 min. After rinsing 3 times with water, the sections were stained with Schiff's Reagent for 15 min, then washed with fresh sulfite water for 5 min. The sections were dehydrated, cleared and mounted. For MSB staining, the paraffin-embedded sections (at 4 µm) were oxidized in 1% potassium permanganate for 5 min, rinsed with water, treated with 2% oxalic acid, stained with Celestine blue for 3 min, and then treated with Mayer' haematoxylin for 3 min. The sections were then washed in tap water for 10 min, rinsed in 95% alcohol, and stained with Martius yellow solution for 30 min. After washing in water, the sections were stained in 1% rosaniline trisulfonic acid for 10 min, washed with 1% phosphotungstic acid for 5–10 minutes or until most of the red dye came out of the connective tissue, and then stained in methyl blue solution for 2 min. Finally the sections were dehydrated, cleared and mounted.

### OVA-induced asthma model

Allergen-induced asthma was induced with eight to nine week old female CC10-Smad7 transgenic and wild type mice sensitized with OVA in alum as previously described[50]. Briefly, mice were sensitized using intraperitoneal injection of 10 µg OVA (Sigma-Aldrich) in 200 µl of PBS, and mixed with 1 mg alum (Serva Electrophoresis, Heidelberg,Germany) on days 1 and 14. From day 21, mice were challenged 30 min/day with aerosolized OVA (1% w/v) for 5 consecutive days. Mice were sacrificed 24 h after the last aerosol challenge. Determination of serum OVA-specific IgE production were conducted as previously described [50].

### Urethane-induced lung cancer model

To induce lung adenoma, eight to nine week old male and female mice (with 50% C57BL/6J and 50% A/J background) were given a single dose of urethane in 0.9% NaCl (1 mg/g body weight) intraperitoneally. The mice were sacrificed 5 months after the injection and the lungs were harvested for analysis.

### Lung homogenate and ELISA

The concentrations of cytokine were measured in lung homogenates by multiplex ELISA (Lincoplex Systems, St Charles, MO, USA) on a Luminex 200 system using Starstation software (Applied Cytometry Systems, Sheffield, UK). Following cytokines were included: IL-1β, IL-2, IL-4, IL-5, IL-6, IL-7, KC (murine homologue of IL-8), IL-9, IL-12 (p70), IL-17, IFN-γ, granulocyte-macrophage colony-stimulating factor (GM-CSF), granulocyte colony-stimulating factor (G-CSF), monocyte chemoattractant protein-1 (MCP-1, also known as JE), macrophage inhibitory protein-1α (MIP-1α), TNF-α, and RANTES. TGF-β1 was determined in lung tissue homogenates using a TGF-β Quantikine Kit according to the instructions of the manufacturer (R&D Systems, Minneapolis, MN, USA).Briefly, 96-well microtiter plates were coated overnight at 4°C with 2 µg per well of mouse capturing mAbs and subsequently blocked at 37°C for 2 hours with 2% BSA-PBS. After washing with a solution containing 0.02% Tween-20, 50 µl of each sample and its control were added and incubated for 2 h at ambient temperature simultaneously with 50 µl of a biotinylated detecting antibody (0.25 µg/ml of each monoclonal antibody) in 2% BSA/PBS/Tween-20. The plates were washed and incubated for 30 min with streptavidin-conjugated horseradish peroxidase. 100 µl of 0.0125% tetramethylbenzidine and 0.008% H_2_O_2_ in citrate buffer was used as substrate. A standard curve was performed for each plate and used to calculate the absolute concentrations of cytokines.

## Supporting Information

Figure S1 (0.14 MB DOC)
