# Supplementary material for: In Vivo Disruption of TGF-β Signaling by Smad7 in Airway Epithelium Alleviates Allergic Asthma but Aggravates Lung Carcinogenesis in Mouse
Source: PLoS One. 2010 Apr 13;5(4):e10149. doi: 10.1371/journal.pone.0010149 (PMC2854155; doi:10.1371/journal.pone.0010149)

**Figure S1. Overexpression Smad7 in lung epithelium causes no significant changes in apoptosis.**

Lung sections from the OVA -sensitized and challenged wild type and CC10-Smad7 transgenic mice were used in TUNEL assay to determine cell apoptosis. Representative sections (400X) are shown and the pretreatment with DNase I (0.3 unit/μl) for 10min was used as positive control.


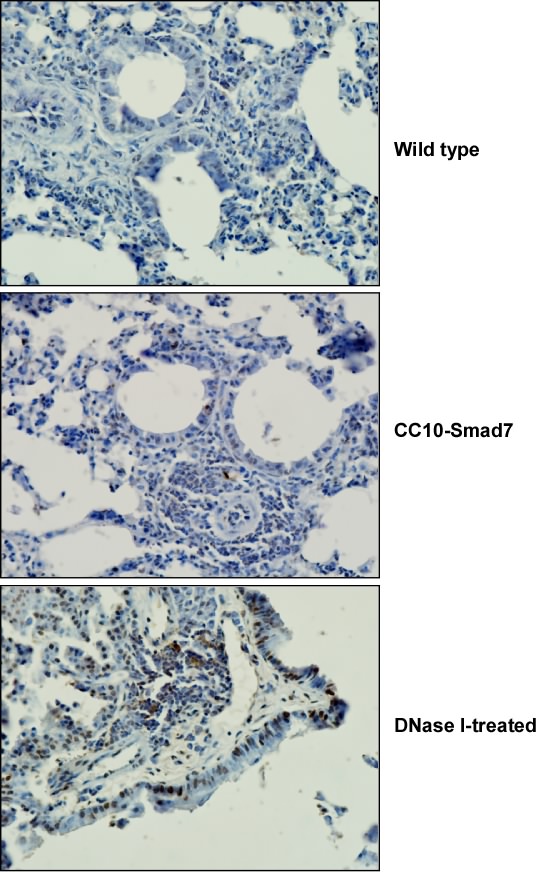

Supplement: Figure S1 — (0.14 MB DOC) [file pone.0010149.s001.doc]
